# DNAJA4 Promotes the Replication of the Chinese Giant Salamander Iridovirus

**DOI:** 10.3390/genes14010058

**Published:** 2022-12-24

**Authors:** Zijing Liu, Daofa Xie, Xianhui He, Tianhong Zhou, Wei Li

**Affiliations:** 1 College of Life Science and Technology, Jinan University, Guangzhou 510642, China; 2 Southern Marine Science and Engineering Guangdong Laboratory (Zhuhai), Guangzhou 510632, China

**Keywords:** DNAJA4, ranavirus, replication, interference, interaction

## Abstract

The DNAJ family, a class of chaperone proteins involved in protein folding, assembly, and transport, plays an essential role in viral infections. However, the role of DNAJA4 (DnaJ Heat Shock Protein Family (Hsp40) Member A4) in the ranavirus infection has not been reported. This study demonstrates the function of the epithelial papilloma of carp (EPC) DNAJA4 in Chinese giant salamander (Andrias davidianus) iridovirus (CGSIV) replication. DNAJA4 consists of 1479 base pairs and encodes a 492 amino acid polypeptide. Sequence analysis has shown that EPC DNAJA4 contains a conserved J domain and shares 84% homology with Danio rerio DNAJA4 and 68% homology with Homo sapiens DNAJA4. EPC DNAJA4 was localized in the cytoplasm, and its expression was significantly upregulated after CGSIV infection. Overexpression of EPC DNAJA4 promotes CGSIV replication and CGSIV DNA replication. siRNA knockdown of DNAJA4 expression attenuates CGSIV replication and viral DNA replication. Overexpression and interference experiments have proved that EPC DNAJA4 is a pro-viral factor. Co-IP, GST–pulldown, and immunofluorescence confirmed the interaction between EPC DNAJA4 and CGSIV proliferating cell nuclear antigen (PCNA). Our results demonstrate for the first time that EPC DNAJA4 is involved in viral infection by promoting viral DNA replication and interacting with proteins associated with viral replication.

## 1. Introduction

Heat shock proteins (HSPs) were initially discovered as a class of acute response proteins in Drosophila melanogaster cultured at high temperatures [1]. Various stress conditions, such as temperature change, ultraviolet radiation, and microbial infection, induce their synthesis [2,3]. HSP is mainly involved in protein folding, non-covalent assembly, and transport of macromolecules, which play a crucial role in maintaining protein homeostasis, cell survival [4,5], and other biological functions [6]. The HSP40/DNAJ family is the most prominent family of human heat shock proteins, and fifty different isoforms have been discovered [7]. DNAJ family proteins contain the conserved J domain [8,9], divided into DNAJA, DNAJB, and DNAJC according to the domain. The DNAJA subclass has the most abundant structure, including a glycine/phenylalanine-rich region (G/F), a cysteine-rich region, and a variable C-terminal domain. Studies have shown that DNAJA4 is widely involved in several virus infections [10]. DNAJA4 in chick bursa is upregulated after Reticuloendotheliosis virus (REV) infection and promotes viral replication [11]. DNAJA4 expression was significantly induced in pigs infected with Salmonella serotype cholerae or HIV [3,12]. Deletion of DNAJA4 after heat shock can enhance NF-kB activation in the HaCaT cells [13]. All of these studies highlight the importance of DNAJA4 in viral infections, and DNAJA4 has the potential to become an emerging therapeutic target in the field of viral research. However, the specific function of DNAJA4 in the process of aquatic virus infection still needs to be clarified, and studies still need to focus on the role of DNAJA4 in ranavirus infection.

Ranavirus is a nucleoplasmic large DNA virus that can be transmitted among ectothermic invertebrates and cause high morbidity and mortality to the infected hosts [14,15], which has caused severe economic and ecological losses to the aquaculture industry worldwide in the past decade [16]. Chinese giant salamander is a second-class protected animal with significant ecological and economic value. With the development of high-density farming and environmental pollution, diseases have gradually become a key factor restricting the healthy development of giant salamander farming [17,18,19,20]. Chinese giant salamander rainbow virus (CGSIV) is a new virus isolated from infected giant salamanders in Zhangjiajie, Hunan Province, which is mainly responsible for the economic loss of the Chinese giant salamander [20]. However, host factors have not revealed the mechanism involved in infection replication. At present, some host factors, such as IRF, Uba2, and MSH2, are involved in virus replication in the studies of ranavirus [21,22,23], but the role of DNAJA4 in ranavirus infection has not been reported. In addition, only the effects of CGSIV MCP on CGSIV infection have been reported [20], and no studies have found that host factors are involved in the replication of CGSIV infection. In this study, the molecular function of EPC DNAJA4 in the replication of CGSIV virus infection was investigated using an in vitro cultured epithelial papilloma of carp (EPC) model. In this study, we identified EPC DNAJA4, investigated its expression and cellular localization in CGSIV infection, and analyzed the role of DNAJA4 in the replication of CGSIV infection.

## 2. Materials and Methods

### 2.1. Viruses, Cells

The CGSIV (Chinese Giant Salamander Iridovirus) and epithelial papilloma of carp (EPC) line used in this study were stored in our laboratory [20]. EPC cells were cultured at 26 ℃ in M199 (M199, BI, Israel) medium supplemented with 10% fetal bovine serum (FBS, Gibco, Australia) [24]. EPC cells were infected with CGSIV with an MOI of 0.1. Virus-infected cells were collected 3 days after infection, frozen and thawed three times, and stored in a liquid nitrogen until use, which was the prepared virus stock solution.

### 2.2. Plasmid Construction

Recombinant plasmids were constructed as described previously [24]. The corresponding primers were used to amplify different types of nucleic acid fragments (Table 1). Plasmids were constructed for overexpression, Co-IP, and GST–pulldown assay. All plasmids used in this study were confirmed by DNA sequencing. Specific operations are as follows:

The full-length open reading frame sequence of EPC DNAJA4 was obtained using PCR amplification from the cDNA obtained by reversing the total RNA of EPC cells. In order to construct the plasmid overexpressing EPC DNAJA4, the coding gene of EPC DNAJA4 was amplified with primers DNAJA4-F1/R1 and cloned into pcDNA3.1-HA to obtain the overexpression plasmid pcDNA3.1-DNAJA4-HA.

Plasmids for co-immunoprecipitation (Co-IP): In order to construct a PCNA plasmid interacting with EPC DNAJA4, the coding gene of CGSIV-PCNA was amplified with primers PCNA-F1/R1 and cloned into pcDNA3.1 to obtain plasmid pcDNA3.1-PCNA-Flag.

Plasmids for GST–pulldown: EPC DNAJA4 was amplified with DNAJA4-F2/R2 and cloned into pET22b plasmid to obtain pET22b-DNAJA4-His. The CGSIV-PCNA encoding gene was amplified into pEGX4T7 plasmid with primers PCNA-F2/R2 to construct a prokaryotic plasmid pEGX4T7-PCNA expressing GST-PCNA protein.

### 2.3. qPCR Was Used to Detect Gene Transcription

The transcription level of EPC DNAJA4 was detected using RT–PCR. EPC cells were infected with CGSIV multiple infection (MOI) of 0.1 or simulated infection, and EPC cells were collected at the specified time after infection. RNA was extracted using TRIzol reagent (Life Technologies, USA) according to the manufacturer’s instructions. RNA was reverse transcribed into cDNA using the Access Reverse Transcription polymerase chain reaction (RT–PCR) System Kit (Promega, USA). RT–PCR analysis was performed with qDNAJA4-F/R primer. The internal control gene 18s was also analyzed by RT–PCR [25,26]. Primer sequences are shown in Table 1.

To analyze the up-regulation and down-regulation of EPC DNAJA4 transcription levels, EPC cells were transfected with overexpression plasmids and siRNA by liposomes in 6-well plates and infected with the virus. The cell virus cultures were collected 24 h after virus infection. Cell viral RNA was extracted. qPCR was used to detect the transcription level of EPC DNAJA4. The reference gene was β-actin or 18s. The expression of target genes was normalized to the reference genes and calculated using the 2^−ΔΔCt^ method. Specific primers are shown in Table 1.

### 2.4. Immunofluorescence Was Used to Detect the Subcellular Localization of the Proteins

To clarify the in vitro subcellular localization of EPC DNAJA4 and CGSIV PCNA, pcDNA3.1-PCNA-Flag and pcDNA3.1-DNAJA4-HA plasmids were transfected into EPC cells using Lipofectamine 2000 reagent (Invitrogen) according to the manufacturer’s instructions. Then, 48 h after transfection, EPC cells were fixed with 4% paraformaldehyde (PFA), treated with PBS containing 1% TritmX-100, blocked with 5%BSA, primary antibody and Alexa Fluor 647 labeled secondary antibody dilution, Hochest 33342 to avoid light staining of nuclear color, and washed with PBS. The samples were examined under a fluorescence microscope (Zeiss, Germany). In addition, to observe the intracellular localization of EPC DNAJA4 after CGSIV infection, EPC cells were placed in 35 mm glass-bottom culture dishes overnight and infected with CGSIV (MOI = 0.1) for 24 h. Indirect immunofluorescence assay (IFA) was performed as described above.

### 2.5. Overexpression and siRNA Knockdown of EPC DNAJA4

When the DNAJA4 plasmid was overexpressed, GFP green fluorescent protein plasmid was used as the reference for transfection efficiency, and StarFect High-efficiency transfection Reagent (GenStar, Beijing, China) was used as the transfection reagent and was transfected according to the proportion specified in the instructions. The transfection efficiency was more than 90%.

Three siRNAs were designed at the front, middle, and back of the DNAJA4 ORF sequence, named siJA4-1, siJA4-2, and siJA4-3. A NC siRNA was designed as a negative control. A FAM-fluorescently labeled siRNA was selected as a reference for transfection efficiency. Synthesized by Shanghai Shenggong Biological Co., LTD. (Sangon Biotech, Shanghai, China). The sequence of DNAJA4 siRNA interference is shown in Table 1. Two siRNA with good effect were selected for the experiment. EPC cells were transfected using PolyHigh transfection (Sangon Biotech, Shanghai, China) at 25 nM. Cells were infected with CGSIV at an MOI of 0.1 and harvested at the indicated time points for RNA or DNA extraction. The targeted siRNA sequences of EPC DNAJA4 mRNA and those targeted by the control RNAi plasmid are shown in Table 1.

### 2.6. CGSIV Viral Titer

To determine the virus yield, virus growth was terminated by freezing virus cultures at the indicated time (48 h for overexpression and siRNA studies). Virions were released by three cycles of freeze–thaw, and cell debris was removed via low-speed centrifugation. After clarification, the virus-containing supernatant was serially diluted 10-fold, and 200 μL of each diluent was added to EPC wells grown on 96-well plates. The viral titer was detected using the TCID50 method.

### 2.7. qPCR Was Used to Detect the Copy Number of Viral Genomic DNA

To measure viral genomic DNA copy number, viral growth was stopped by freezing cultures at the indicated times (overexpression: 1 h, 3 h, 6 h, and 16 h). Virions were released by three cycles of freeze–thaw, and cell debris was removed by low-speed centrifugation. Viral genomes were extracted using the Viral Genome Extraction Kit (omega, Norcross, USA). Using the pMD18T-MCP plasmid as the standard, CGSIV MCP gene primers were designed to evaluate CGSIV DNA replication [22]. qMCP-F/R was used as upstream and downstream primers, and the copy number of viral genomic DNA was determined by absolute quantitative PCR on an ABI 7300 PCR instrument using Takara SYBR^®^ Premix Ex Taq™ II.

### 2.8. Protein Interaction Assay

Co-IP experiment: Eukaryotic inducible expression plasmids were constructed and co-transfected into EPC cells, and the cells were lysed with RIPA lysate 48 h after transfection for co-immunoprecipitation and Western blotting analysis as previously described [27].

GST–pulldown experiment: Gst-pcna-bound glutathione agarose beads were incubated for 3 h with Rosetta bacterial lysate containing DNAJA4-His protein. The sample was first washed three times with RIPA lysis buffer, then mixed with an equal volume of 2×SDS loading buffer and boiled for 10 min. Immunoblot analysis was performed as previously described [28]. 

### 2.9. Statistical Analysis

All experiments in this study were performed in triplicate independently, and the data are expressed as mean ± standard deviation (mean ± SD). GraphPad Prism 7 software was used for the statistical analysis of experimental data, and an independent sample *t*-test (Student’s *t*-test, *p* < 0.05) was used for comparison between groups (two-group comparison), as well as one-way ANOVA followed by Bonferroni post hoc test (multigroup comparison). *p* < 0.05 indicates that the difference between groups is statistically significant (* *p* < 0.05, ** *p* < 0.01, *** *p* < 0.001, **** *p* < 0.0001); ns indicates no significant difference.

## 3. Results

### 3.1. Sequence Characterization of EPC DNAJA4

**Figure 1:**
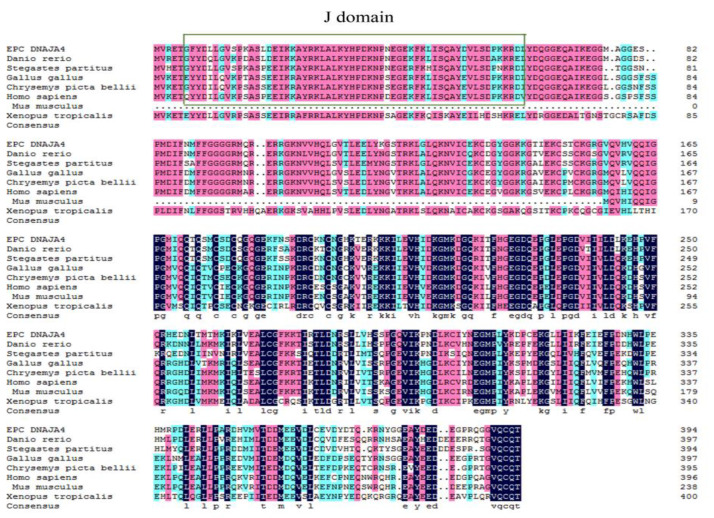
Amino acid alignment of DNAJA4s from different species. The conserved domains (aa 0–70), respectively. The accession numbers were listed as follows: Epinephelus coioides, MN937271; Danio rerio, XP_002666797.3; Stegastes partitus, XP_008275021.1; Gallus gallus, XP_413746.5; Chrysemys picta bellii, XP_005294547.1; Homo sapiens, AIC63476.1; Mus musculus, AAH22948.1; Xenopus tropicalis.

The full-length cDNA of EPC DNAJA4 was obtained using PCR amplification according to the EST sequence of the EPC transcriptome. The cDNA of EPC DNAJA4 is 1188 bp in length and encodes a 395 amino acid polypeptide, showing 84% and 68% homology with zebrafish DNAJA4 and human DNAJA4 orthologs, respectively. As shown in Figure 1, EPC DNAJA4 contains a conserved J domain at amino acids 5–60. The 79–95aa of EPC DNAJA4 contains a glycine/phenylalanine-rich region (G/F domain), 133–197aa contains a conserved cysteine-rich region (zinc-finger domain), and 215–395aa contains a C-terminal domain. The prediction of the nuclear entry signal showed that DNAJA4 at amino acids 20–50 and 342–374 might be the nuclear entry signal region of NLS, suggesting that DNAJA4 may be able to shuttle through nucleoplasmic regions under certain conditions.

**Figure 2:**
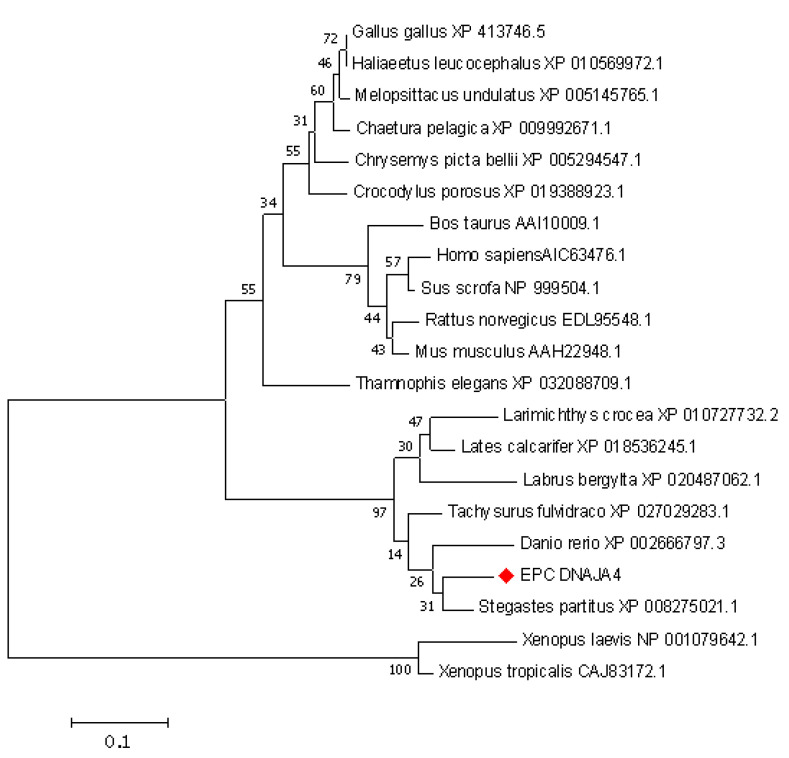
Phylogenetic analysis of DNAJA4 homologs from different species. A phylogenetic tree was constructed based on the protein sequences of DNAJA4-like genes from different species using MEGA 7.0 with the neighbor-joining (NJ) method. Numbers at the nodes denote the bootstrap values of 1000 replicates. Scale represents the numbers of substitutions per 1000 bases.

The results of the cluster analysis are shown in Figure 2. Phylogenetic analysis showed that it clustered into two clades: teleost fish, mammals, and birds clustered into one group, and, intriguingly, amphibians formed a relatively separate clade. EPC DNAJA4 was classified as a teleost fish, and its amino acid homology was most closely related to Danio rerio (84.42%), followed by Tachysurus fulvidraco, Stegastes partitus, and other species (Figure 2).

### 3.2. Expression and Localization of EPC DNAJA4

**Figure 3:**
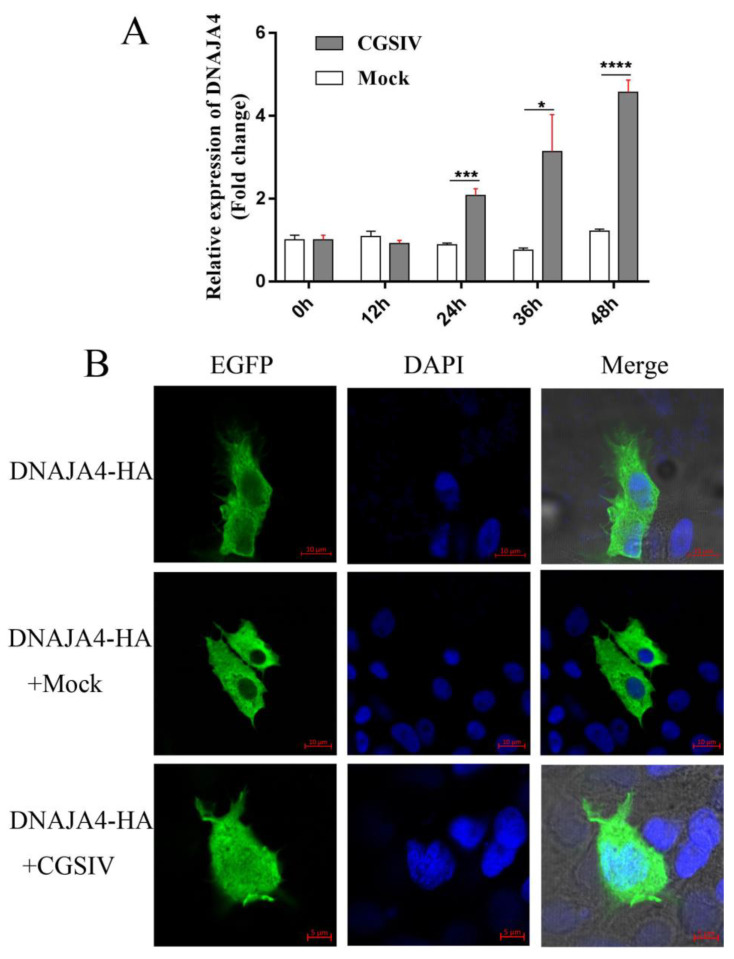
Viral infection affected the expression and localization of EPC DNAJA4. (**A**) Effect of viral infection on EPC DNAJA4 transcription levels; (**B**) effects of viral infection on the localization of EPC DNAJA4. The results were expressed as mean ± SD, (*n* = 3; * *p* < 0.05; *** *p* < 0.001; **** *p* < 0.0001).

The transcriptional differences of DNAJA4 cells at different times after CGSIV infection were detected. As shown in Figure 3A, within 0–48 h of viral infection, the transcript level of DNAJA4 increased with the extension of viral infection time compared with that of the mock group (inactivated CGSIV), and the peak value was 3.75 times that of the mock group, with a statistically significant difference (*p* < 0.0001). The results showed that CGSIV infection upregulated the expression of EPC DNAJA4.

To explore the subcellular localization of EPC DNAJA4 in vitro, pcDNA-DNAJA4-HA was transfected into EPC cells and observed under a laser confocal microscope after immunofluorescence staining. As shown in Figure 3B, the green fluorescence of EPC DNAJA4 was distributed in the cytoplasm. Therefore, EPC DNAJA4 is presumed to be a cytoplasmic protein. Immunofluorescence was used to further investigate the localization of EPC DNAJA4 after virus infection. In the mock group, green fluorescence was distributed in the cytoplasm. In the CGSIV infection group, the distribution of green fluorescence was changed, most of the green fluorescence existed in the cytoplasm, and some of the green fluorescence existed in the nucleus. We observed this in 70% of the infected cells. The results indicated that the localization of EPC DNAJA4 changed after virus infection, and some EPC DNAJA4 entered the nucleus from the cytoplasm.

### 3.3. Overexpression of EPC DNAJA4 Enhanced CGSIV Replication

**Figure 4:**
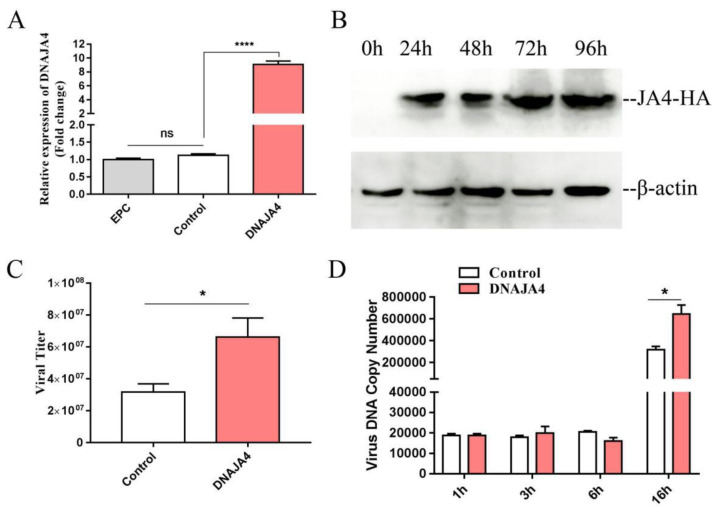
Effect of overexpression of DNAJA4 on CGSIV replication: (**A**) transcriptional level detection 24 h after overexpression of pcDNA3.1-DNAJA4-HA plasmid; (**B**) detection of protein level of plasmid overexpressing pcDNA3.1-DNAJA4-HA; (**C**) effect of overexpression of DNAJA4 for 48 h on viral titer; and (**D**) effect of overexpression of DNAJA4 on the copy number of viral genomic DNA. The results were expressed as mean ± SD, (*n* = 3; * *p* < 0.05; **** *p* < 0.0001).

To investigate the role of EPC DNAJA4 in CGSIV replication, we examined the transcription and protein profile of the plasmid overexpressing pcDNA3.1-DNAJA4-HA. As shown in Figure 4A, the transcription level of DNAJA4 in the DNAJA4 overexpression group was 9.1 times higher than that in the negative control and blank control group (*p* < 0.0001), and there was no significant difference in the transcription level of DNAJA4 between the negative control and blank control group. As shown in Figure 4B, the protein expression of DNAJA4-HA was already expressed 24 h after transfection and continued to be expressed until 96 h after transfection.

The viral titer results are shown in Figure 4C. The average viral titer in the transfected pcDNA3.1-DNAJA4-HA plasmid overexpressing DNAJA4 group was 6.62 × 10^7^ TCID50/mL, and that in the transfected pcDNA3.1-HA negative control group was 3.18 × 10^7^ TCID50/mL. The average viral titer in the DNAJA4 overexpression group was significantly higher than that in the negative control group, and there was a statistically significant difference in the average viral titer between the two groups by *t*-test (*p* < 0.05, *p* = 0.0103). The results showed that overexpression of EPC DNAJA4 enhanced both CGSIV replication in vitro and the production of infected progeny CGSIV virions.

The viral genomic copy number results are shown in Figure 4D. There was no difference in the copy number of viral genomic DNA between the DNAJA4 overexpression group and the negative control group at 1 h, 3 h, and 6 h after CGSIV infection, indicating that EPC DNAJA4 overexpression did not promote CGSIV DNA replication at the early stage of virus infection. Overexpression of EPC DNAJA4 had no effect on viral genomic DNA replication between 1 h and 6 h after CGSIV infection (Figure 4D). However, the average copy number of viral genomic DNA in the overexpression group and the control group at 16 h after virus infection was 6.45 × 10^6^ and 3.19 × 10^6^, respectively. The average viral genomic DNA copy number in the DNAJA4 overexpression group was significantly higher than that in the negative control group, and there was a statistically significant difference in the average viral genomic DNA copy number between the two groups by *t*-test (*p* < 0.05, *p* = 0.013). The results showed that overexpression of EPC DNAJA4 enhanced CGSIV DNA replication.

### 3.4. Knockdown of DNAJA4 Attenuated CGSIV Replication

**Figure 5:**
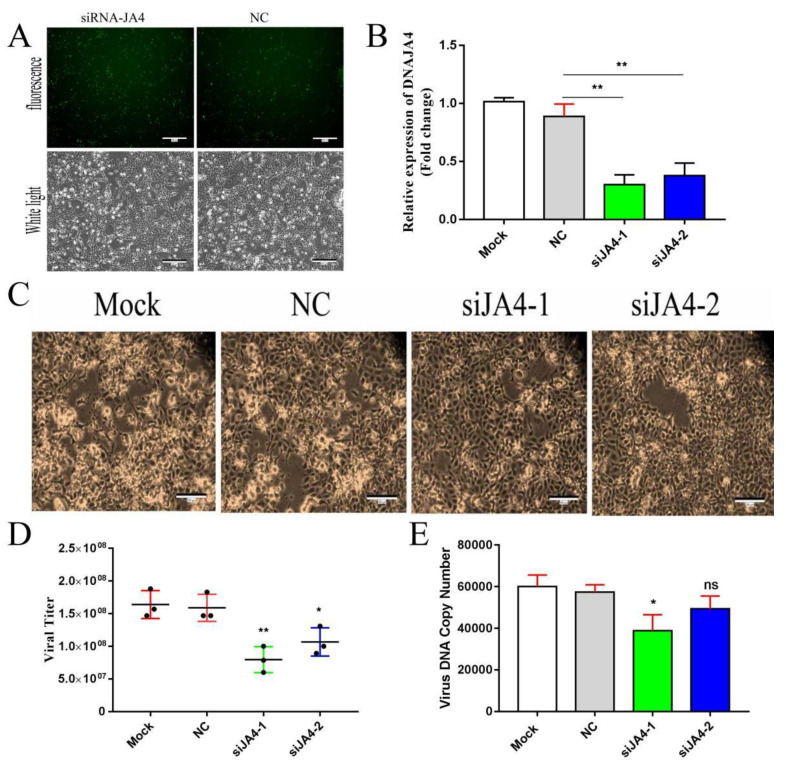
Knockdown of DNAJA4 by siRNA attenuates CGSIV replication and CGSIV DNA replication: (**A**) transfection efficiency of fluorescent siRNA; (**B**) qPCR detection of siRNA interference effect; (**C**) results of siRNA interference with CPE; (**D**) effect of siRNA interference on the titer of CGSIV virus; and (**E**) effect of siRNA interference on genome replication of CGSIV virus. The results were expressed as mean ± SD, (*n* = 3; * *p* < 0.05; ** *p* < 0.01).

The transfection efficiency of siRNA was evaluated according to the fluorescence coverage ratio of fluorescent siRNA. As shown in Figure 5A, green, fluorescent siRNA basically covered all adherent cells, and the transfection efficiency was relatively high. DNAJA4 mRNA levels were detected by RT–qPCR to evaluate the interference efficiency of siRNA, as shown in Figure 5B, and compared with the mock group, siJA4-1 and siJA4-2 sets of DNAJA4 mRNA were significantly reduced by 70% and 63%, with a significant difference in statistics (*p* < 0.01, *p* = 0.0017, *p* = 0.0043). The results indicated that siJA4-1 and siJA4-2 could effectively knock down the transcription level of DNAJA4 in EPC cells 48 h after transfection. After 36 h of siRNA interference with DNAJA4, the phenomenon of viral CPE was observed via fluorescence microscopy. As shown in Figure 5C, the degree of CPE infection in siJA4-1 and siJA4-2 groups was significantly lower than that in the NC control group.

The viral titer results of siJA4-1 and siJA4-2 knockdown are shown in Figure 5D. The average viral titer of the siJA4-1 group was 7.96 × 10^7^ TCID50/mL, and that of the siJA4-2 group was 1.07 × 10^8^ TCID50/mL. The average viral titers of the NC group and blank control group were 1.59 × 10^8^ TCID50/mL and 1.64 × 10^8^ TCID50/mL, respectively. The average viral titers in the siJA4-1 and siJA4-2 groups were about 50% and 33% lower than those in the NC group, respectively. There was a statistically significant difference in the average viral titers in the siJA4-1 and siJA4-2 groups compared to those in the NC group by *t*-test (*p* = 0.009, *p* = 0.039, *p* < 0.05). The results showed that the knockdown of DNAJA4 by siRNA attenuated CGSIV replication.

As shown in Figure 5E, the average viral genomic DNA copy numbers of siJA4-1 and siJA4-2 groups were 3.89 × 10^4^ and 4.94 × 10^4^, respectively. The average viral genomic DNA copy numbers of the NC transfected group and blank control group were 5.74 × 10^4^ and 6.00 × 10^4^, respectively. The average viral genomic DNA copy number of the siJA4-1 and siJA4-2 groups decreased by 30% and 14%, respectively, to levels which were lower than those of the NC group and the control group. There was a statistically significant difference between the siJA4-1 group and NC group by *t*-test (*p* = 0.019, *p* < 0.05). There was no significant difference between the siJA4-2 group and NC group (*p* = 0.12). The results showed that the knockdown of DNAJA4 expression by siRNA attenuated CGSIV DNA replication.

### 3.5. EPC DNAJA4 Interacts with CGSIV PCNA

Overexpression and knockdown experiments showed that EPC DNAJA4 could promote viral replication. However, there are many mechanisms by which DNAJA4 promotes CGSIV replication, and some studies have found that DNAJA4 may interact with proteins related to viral DNA replication. Viral PCNA is a progressive factor of viral DNA polymerase replication, which plays the role of polymerase switch factor and recruitment factor in the process of viral DNA replication, participates in the synthesis of the nascent strand, and plays an essential role in the process of viral DNA repair and recombination. To investigate the viral proteins interacting with EPC DNAJA4, CGSIV PCNA was demonstrated to interact with EPC DNAJA4 by Co-IP, GST–pulldown, and immunofluorescence localization.

**Figure 6:**
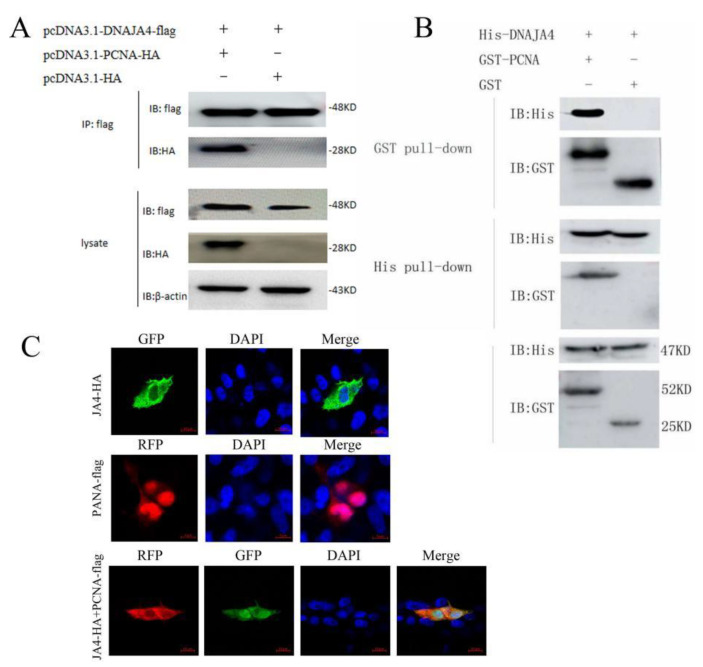
Interaction between EPC DNAJA4 and CGSIV PCNA: (**A**) Co-IP detects the interaction between EPC DNAJA4 and CGSIV PCNA. CO-IP was immunoblotted with anti-Flag antibody and anti-HA antibody. (**B**) His–pulldown and GST–pulldown verified that EPC DNAJA4 interacts with CGSIV PCNA. (**C**) Immunofluorescence detection shown of DNAJA4-HA and PCNA-Flag co-localization in EPC.

Co-IP results are shown in Figure 6A, and both PCNA-Flag and DNAJA4-HA fusion proteins were overexpressed in EPC cells. After the incubation of protein-A/G beads with the anti-Flag antibody, the full-length DNAJA4-HA protein could be detected. The results showed that CGSIV PCNA and DNAJA4-HA protein could interact in the cell.

The results of the His–pulldown experiment showed in Figure 6B that the expressed DNAJA4-his protein, GST-PCNA protein, and GST protein were incubated at the same time. After incubation of agarose beads with ANTI-His antibody, the GST-PCNA protein could be detected using western blot with GST antibody. However, the GST protein was not detected. The results showed that the DNAJA4-His protein could bind to the GST-PCNA protein. GST–pulldown test results showed that the GST-PCNA protein, GST protein, and DNAJA4-His protein were incubated at the same time, agarose GST beads were incubated with ANTI-GST antibody. DNAJA4-His incubated with the GST-PCNA protein could be detected, while DNAJA4-His incubated with the GST protein was not detected. The results showed that the GST-PCNA protein could bind to the DNAJA4-His protein, but the GST protein could not bind to DNAJA4-His.

The co-localization of PCNA and DNAJA4 in cells was studied using the confocal method. IFA results in Figure 6C show that EPC DNAJA4-HA was expressed alone, and the green fluorescence was distributed in cytoplasm and nucleus. The virus PCNA-Flag protein was expressed alone, and the red fluorescence was localized in the nucleus and cytoplasm. When DNAJA4-HA protein and PCNA-Flag protein were co-expressed, DNAJA4-HA red fluorescent protein was localized in the cytoplasm, and PCNA-Flag green fluorescent protein was localized in the cytoplasm and nucleus. Red and green fluorescence produced orange superimposed fluorescence in the cytoplasm. The results showed that the DNAJA4 protein co-localized with the viral PCNA protein in the cytoplasm.

## 4. Discussion

DNAJA4 is involved in many biological processes in mammals, including immunity, development, disease, and therapy [29,30,31]. In addition, studies have shown that DNAJA4 also plays a crucial role in viral infections [32,33]. However, the functional role of DNAJA4 in ranavirus infection has not been reported. Ranavirus has a high lethality in infected hosts and can be transmitted among ectothermic invertebrates [34], leading to substantial economic losses to the aquaculture industry worldwide [16]. Chinese giant salamander iridovirus (CGSIV), a member of the ranavirus genus, causes high morbidity and mortality in cultured and wild Chinese giant salamanders [20]. Since the mechanism of CGSIV infection and replication is still unclear, and there are no effective prevention and control measures for ranavirus, including CGSIV, it is particularly urgent to study the influence and interaction of host factors on CGSIV infection and replication. In the early stage of this study, host proteins interacting with CGSIV PCNA were screened by yeast two-hybridization, including DNAJA4. In this study, a homolog of DNAJA4 was cloned from EPCs, and the molecular characteristics of EPC DNAJA4 were identified. The expression and localization of EPC DNAJA4 before and after virus infection, its effect on CGSIV replication, and its interaction with CGSIV replication-related proteins were investigated.

Sequence analysis of Figure 1 shows that the cDNA of EPC DNAJA4 encodes a 395 amino acid polypeptide with a conserved J domain of heat shock protein at amino acids 5–60, which is highly conserved in zebrafish, hidden chicken, Mediterranean rock dragon, tropical Claus frog, Homo sapiens, and mice. This analysis is consistent with Liu’s research that the J domain of HSP is conserved from fish to mammals [9]. Based on the results of the evolutionary tree, EPC DNAJA4 is closely related to DNAJA4 of zebrafish, and Tachysurus fulvidraco and DNAJA4 may have similar functions in these species. Evolutionary analysis showed that amphibian DNAJA4 formed another relatively independent branch. The structure and function of DNAJA4 as a stress response protein in amphibians were more diverse, possibly because amphibians had more abundant stimuli in aquatic and land environments. Immunofluorescence results showed that EPC DNAJA4 was distributed in the cytoplasm of EPCs, which was different from the distribution of human DNAJA4 subcellular localization from the cell membrane to the nucleus [35]. These results suggest that EPC DNAJA4 may have both similar and different functions than mammalian DNAJA4. CGSIV infection significantly upregulated the transcription level of EPC DNAJA4 and changed its localization in host cells; that is, most of the fluorescence was localized in the cytoplasm, and some were in the nucleus. This is in line with the prediction of the nuclear entry signal that DNAJA4 at amino acids 20–50 and 342–374 may be NLS’s nuclear entry signal regions. These results suggest that DNAJA4 may play a related role in virus infection by translocation into the nucleus. Studies have shown that NbDnaJ can participate in potato virus X (PVX) replication and traction virus movement [36]. It is speculated that DNAJA4 may be involved in the transfer of the CGSIV virus from the nucleus to the cytoplasm, thereby promoting DNA replication in the later stages of virus infection.

As shown in Figure 4A, the plasmid of EPC DNAJA4 effectively upregulated the mRNA level of DNAJA4 in the EPCs by up to 9.1 times. As shown in Figure 4C, the viral titer results showed that overexpression of EPC DNAJA4 promoted CGSIV replication and the production of infectious progeny virions. Further study of viral DNA copy number showed that overexpression of EPC DNAJA4 did not promote CGSIV DNA replication in the early stage of viral infection (0–6 h) but promoted CGSIV DNA replication at 16 h of viral infection (Figure 4D). The results suggest that EPC DNAJA4 may play a role in the packaging and assembly of virions in the later stage of virus infection and promote the replication of CGSIV DNA by promoting the replication of CGSIV DNA. siRNA interference effectively knocked down DNAJA4 mRNA levels to 30% in EPCs (Figure 5B). As shown in Figure 5C and Figure 5D, knockdown of EPC DNAJA4 expression could attenuate CGSIV viral titer and CGSIV DNA replication, suggesting that knockdown of EPC DNAJA4 expression might attenuate CGSIV virus replication by attenuating CGSIV DNA replication. Studies have shown that DNAJA1 enhances influenza A virus RNA polymerase activity and increases viral RNA synthesis in vivo and in vitro [37]. These results suggest that DNAJA4 may also promote CGSIV DNA replication by enhancing the activities of CGSIV replication-related enzymes. In addition, we found that overexpression of DNAJA4 promoted the transcription of viral genes and inhibited the transcription of host cell immune factors IRF3 and IFN, suggesting that DNAJA4 promoted the diversity of CGSIV replication mechanisms. Studies have demonstrated that DNAJ can inhibit HBV replication and effectively activate HSV-1 and HPV DNA replication [38,39,40], indicating the diversity of DNAJ protein functions and the different roles of DNAJ protein in different virus infections.

Co-IP obtained CGSIV PCNA protein interacting with EPC DNAJA4, and GST–pulldown confirmed the direct interaction between EPC DNAJA4 and CGSIV PCNA. Immunofluorescence was used to demonstrate that EPC DNAJA4 co-localized with CGSIV PCNA in the cytoplasm. Studies have found that human DnaJ protein can interact with HSV-1 replication-related UL9 protein to promote viral DNA replication [39]. PCNA is also a replication-related protein [41,42]. PCNA can surround DNA, slide along it, immobilize DNA polymerases and other DNA editing enzymes, and promote DNA replication [43,44]. Because EPC DNAJA4 functions as a chaperone protein, EPC DNAJA4 may promote CGSIV replication by helping CGSIV PCNA form favorable spatial structures. Studies have shown that RSIV PCNA, as the core gene of ranavirus, is involved in the virus replication compartment [45]. These results suggest that EPC DNAJA4 may also assemble the CGSIV replication compartment with CGSIV PCNA to promote virion packaging and, thus, viral replication. Co-expression of EPC DNAJA4 and CGSIV PCNA changed the main localization distribution of CGSIV PCNA, which translocated CGSIV PCNA from the nucleus to the cytoplasm, which is the replication region of CGSIV. These results suggest that DNAJA4 as a chaperone protein may promote viral replication by promoting the translocation of CGSIV PCNA from the nucleus to the cytoplasm.

## 5. Conclusions

In summary, we cloned the EPC DNAJA4 gene from EPCs and characterized its response to CGSIV. The EPC DNAJA4 gene was localized to the cytoplasm, and its expression was upregulated after EPCs were infected with CGSIV. Overexpression of EPC DNAJA4 promoted CGSIV infection, while its knockdown attenuated CGSIV infection. EPC DNAJA4 interacts with CGSIV PCNA. Our study provides novel insights into regulating the EPC DNAJA4 response to CGSIV infection.

## Figures and Tables

**Table 1:** Sequence of primers used in this study.

Primer Name	Primer Sequence
DNAJA4-F1	CCCAAGCTTGCCACCATGGTTCGAGAAACCGG
DNAJA4-R1	GCTCTAGATTAAGCGTAGTCTGGGACGTCGTATGGGTATTGAGTCTGGCACTGGACAC
DNAJA4-F2	GGGTTTCATATGCACCACCACCACCACCACATGGTTCGAGAAACCGGTTTCTACG
DNAJA4-R2	CCCAAGCTTTTGAGTCTGGCACTGGACACCACCTTGCC
PCNA-F1	CGGAATTCGCCACCATGCTGTGGGAAGCCGTA
PCNA-R1	GCGTCGACTTAGCCCTCAAAGAGAGTCACG
PCNA-F2	CGGAATTCATGCTGTGGGAAGCCGTAACA
PCNA-R2	GATGCGGCCGCTTAGCCCTCAAAGAGAGTCACGGTC
qDNAJA4-F	AAGGCTCAACGAGGAAAC
qDNAJA4-R	CACATGCTCTGGGTCTGC
DNAJA4-80F	GCAAACUGGCAUUGAAAUATT
DNAJA4-80R	UAUUUCAAUGCCAGUUUGCTT
DNAJA4-536F	GCCAAGGACAAGGAGAGAATT
DNAJA4-536R	UUCUCUCCUUGUCCUUGGCTT
DNAJA4-930F	CAAAGACCCUUGUGAGAAATT
DNAJA4-930R	UUUCUCACAAGGGUCUUUGTT
FAM NC-F	UUCUCCGAACGUGUCACGUTT
FAM NC-R	ACGUGACACGUUCGGAGAATT
β-actin-F	CACTGTGCCCATCTACGAG
qMCP-F	CTGGAGAAGAAGAATGGGAGGGG
qMCP-R	CTTTCGGGCAGCAGTTTTCGGTC
β-actin-R	CCATCTCCTGCTCGAAGTC
q18S-F	ATGGTACTTTAGGCGCCTAC
q18S-R	TATACGCTATTGGAGCTGG

## Data Availability

The CGSIV sequence used in the study has been submitted into GenBank under accession number KF512820.1.
